# Attitude of health workers to the care of psychiatric patients

**DOI:** 10.1186/1744-859X-8-19

**Published:** 2009-08-23

**Authors:** Aghukwa Nkereuwem Chikaodiri

**Affiliations:** 1 Department of Psychiatry, Aminu Kano Teaching Hospital, Kano, Nigeria

## Abstract

**Background:**

In a few months from the time of this report, wards for inpatient care of psychiatric patients at the Bayero University Medical School Aminu Kano Teaching Hospital will be ready for admissions. The attitude of staff to the care of such patients within the hospital was the focus of this study.

**Methods:**

The investigation was a descriptive and cross-sectional study on a stratified and randomly selected sample population of workers at the Aminu Kano Teaching Hospital. A questionnaire was used to elicit responses from the respondents, containing 11 modified items. Statistical analysis of responses was performed.

**Results:**

The number of properly completed questionnaires analysed was 362. The result showed that 232 (64.1%) respondents would be fearful of having psychiatric patients admitted within the hospital. In all, 192 (53.0%) would not want their place of work to be next door to the psychiatric wards. Gender showed a significant association with responses on many of the questionnaire items (*P *< 0.05), with more females than males expressing unfavourable attitudes. Profession of the respondents was significantly related to both not wanting ones place of work to be next door to the psychiatric wards and having good reason to resist the location of psychiatric wards within the hospital (*P *< 0.05).

**Conclusion:**

Health workers expressed fears about treating psychiatric patients within a general hospital environment and preferred segregation of the wards and the patients if treated within such a setting. Expansive enlightenment programmes and positive contacts with psychiatric patients during treatment could help reduce stigma to mental illness by health workers.

## Introduction

The propensity of psychiatric patients to cause injury or harm to others and to property is one of the strong stereotype beliefs the Nigerian public holds towards psychiatric patients [1-4]. Psychiatric patients, especially vagrant psychotics, are seen as worthless, dirty, senseless, dangerous and unpredictable [1,4]. Moreover, it is the belief of most people in Nigerian society that psychiatric illnesses are afflictions caused by supernatural forces and, as such, require care by traditional and syncratic religious healers, rather than orthodox care [3].

Most Nigerians judge the seriousness of psychiatric illness on behavioural grounds [1]. Therefore, most deviant behavioural manifestations in society equate to psychiatric illness presentation in the involved person. Many African societies believe that psychiatric illness is either the outcome of an abominable familial defect or the 'handiwork of evil machinations' (demons, evil spirits). Therefore, these negative beliefs result in psychiatric patients being seen as outcasts and people that should be quarantined [3].

The psychiatric patient, in the minds of the Nigerian public, is responsible for their illness, especially when it is an alcohol and/or substance-related problem. This belief denies them the sympathy and understanding traditionally bestowed on the sick in African society [3,4].

Misunderstanding of psychiatric illnesses by the public as outlined above robs the psychiatric patient of, among other things, provision of satisfactory mental and physical healthcare services, even in the tradocultural context. Generally, misconceptions about psychiatric patients being under the control of evil spirits (and therefore being dangerous) are the main motivations behind the inglorious, long and persistent use of physical restraints and endorsing of segregating attitudes by society towards them [5]. Health workers are not completely free from these above-mentioned unfavourable beliefs and attitudes towards psychiatric patients [3,5,6].

The present study, which determined health workers opinions about caring for psychiatric patients within a general hospital setting, took place in Aminu Kano Teaching Hospital, Kano, Nigeria. The Teaching Hospital, established in 1988 for Bayero University Medical School, has state of the art facilities. It is a fully functional, almost 1,000-bed hospital designed to cater for the health needs of communities within the northwestern zone of the country and beyond. In addition, teaching and research in health-related matters are among the services undertaken in the hospital.

Currently, there are 17 clinical departments offering patient services and conducting training and research, as well as over 10 support service departments. The psychiatry department building is among new structural facilities presently been erected within the hospital. Since the foundation of the hospital, inpatient psychiatric services were located at a facility 60 km from the hospital complex. On completion of the new psychiatry department, inpatient psychiatry care would commence within the hospital complex.

The present study, while determining the attitudes of health professionals to the care of psychiatric patients within the hospital, also looked at possible relationships between some of their attitudes to such care and their sociodemographic characteristics. Findings from this study may be a good guide when planning mental health enlightenment programme goals that have hospital workers as the target population.

## Methods

The psychiatry department, within months of this report, will have wards ready for admission of patients within the hospital. The study therefore determined if the attitudes of the health workers at Aminu Kano Teaching Hospital are favourable towards the care of psychiatric patients within the hospital. The study was descriptive and cross-sectional, conducted between February and March 2009.

Samples for the study came from the staff population of the Aminu Kano Teaching Hospital, grouped into nine strata by profession. The sample size of 326 with a 95% confidence interval and a 4.6% margin of error were taken from a population of 1,211 health workers comprising: 253 doctors, 437 nurses, 28 pharmacists, 163 administrators, 113 laboratory scientists, 17 social workers, 17 physiotherapists, 82 medical records officers, and 103 hospital support staff. Due to possible attritions, an additional 10% of the original sample size was added to give a working sample size of 363. The sample comprised 77 medical doctors, 132 nurses, 8 pharmacists, 49 administrators, 34 laboratory scientists, 5 social workers, 6 physiotherapists, 25 medical records officers, and 31 support staff.

The instrument for the study was an 11-item modified and pretested questionnaire, used in a previous community survey on attitudes to mental illness [7]. Since the study sample was from a Nigerian literate population, the questions were in English.

The questionnaire was pretested on 10 conveniently selected staff from the hospital, comprising 3 doctors, 1 medical laboratory scientist, 4 nurses, and 2 hospital administrators. They commented on (1) their ability to understand the questions, (2) what they felt the questions wanted to find out, and (3) any questions they felt needed clarifying, with suggestions on how this could be achieved. There were minor revisions performed on the questionnaire based on the responses from the pilot study.

Focus group discussions on the 11 items in the questionnaire took place among the hospital workers, interdepartmentally at different times. The groups numbered between five to eight members, comprising doctors, nurses, administrators and support staff present at the time of the discussion.

The participants, after going through the questions, expressed their opinions on the topics, while the researcher, who was also the group facilitator, listened and noted their comments on each of the items.

The questions measured the anticipated fear and exclusion intentions of the health workers towards psychiatric patients. Questions asked in the interview focused on: (a) demographic characteristics of the respondents such as age, sex, department, profession, marital status, religion and tribe; and (b) respondents' fear intentions and exclusion reactions towards the admission of psychiatric patients within the hospital.

Some of the statements discussed/questions asked were: 'locating psychiatric wards within the hospital premises does not put other patients on admission in danger', 'it is fearful to think of people with mental problems admitted within the hospital premises', and 'psychiatric wards should be placed outside the hospital premises'.

The respondents' either agreed or disagreed with the questions/statements. A positive or favourable response was one that supports caring for psychiatric patients within the hospital premises. Trainee psychiatrists helped to distribute the questionnaire interdepartmentally. Members of staff in the chosen professions in each department, who picked numbers by balloting, completed the questionnaire. The hospital's ethical committee granted consent to conduct the survey before it commenced.

### Data analysis

Data summary after analysing with the SPSS statistical package (SPSS, Chicago, IL, USA) was with simple frequency distribution tables. Tests of association between some of the responses and some of the respondents' sociodemographic features such as type of profession, gender, and sex were determined with the χ^2 ^test. A *P *value of ≤ 0.05 was considered statistically significant.

## Results

The number of analysed questionnaires that were properly completed was 362, representing 99.7% of the estimated sample size for the study. The recipients comprised 77 (21.3%) doctors, 132 (36.5%) nurses, 8 (2.2%) pharmacists, 49 (13.5%) administrators, 34 (9.4%) laboratory scientists, and 5 (1.4%) social workers, 6 (1.7%) physiotherapists, 31 (8.6%) support staff, and 20 (5.5%) records officers.

Among these, 197 (54.4%) were males and 164 (45.3%) were females; 239 (66.0%) were married, and 119 (32.9%) were single. Three (0.8%) of the respondents were divorced and one (0.3%) was widowed.

The majority (289 (79.8%)) of the respondents were Moslems and 72 (19.9%)) were Christians. The predominant ethnic nationality of the recipients was Hausa (265 (73.2%)), 33 (9.1%) were Igbos, 30 (8.3%) Yorubas, and 34 (9.4%) were from various ethnic minority tribes in the country. The mean age of the respondents was 33.2 ± 5.8, range 20 to 58 years.

More than half (214 (59.1%)) of the respondents were of the opinion that locating psychiatric wards within the hospital premises would not put other patients on admission in danger. In the same manner, 198 (54.7%) of them did not think it was reasonable for the hospital staff to resist the location of psychiatric wards within the hospital. However, 232 (64.1%) would feel fearful at the thought of having people with mental problems admitted within the hospital. A total of 200 (55.2%) respondents felt hospital staff had nothing to fear from psychiatric patients coming to receive treatment within the hospital.

About three in every five (61.0%) respondents' did not think that having psychiatric patients on treatment within the hospital was too great a risk to other people in the hospital. In addition, 299 (82.6%) of the respondents did not think locating psychiatric wards within the teaching hospital would downgrade it.

More than half (192 (53.0%)) of the recipients would not wish to have their place of work next door to the psychiatric wards. Despite the above opinion, 222 (61.3%) did not favour placing psychiatric wards outside the hospital complex.

A total of 276 (76.2%) respondents agreed the location of psychiatric wards should be in the hospital, to best serve the needs of the community. In a similar manner, 239 (66.0%) of them felt nobody had the right to exclude psychiatric wards from being a type of ward located within a teaching hospital.

Many (240 (66.3%)) respondents preferred wards for the admission of psychiatric patients to wards for admission of immune depressed patients (that is, HIV patients).

There were significant associations between the gender of the respondents, and their attitudes to caring for psychiatric patients within the hospital premises. The female respondents held more unfavourable attitudes on most of the items assessing fear and exclusion intentions than the male respondents did (Table 1).

**Table 1:** Crosstabulation report between gender of respondents and their responses on some variables assessing perceived fear towards psychiatric patients

Variable	Male		Female				
				
	Agree (%)	Disagree (%)	Agree (%)	Disagree (%)	χ^2^	df	*P *value
Not a danger to other patients	129 (65.5%)	68 (34.5%)	84 (51.2%)	80 (48.8%)			
Good reason to resist location	73 (37.1%)	121 (61.4%)	88 (53.7%)	76 (46.3%)	12.6	4	0.01
Too great a risk to other people	61 (31.0%)	136 (69.0%)	79 (48.2%)	84 (51.2%)	13.4	4	0.01
Wards should be placed outside the hospital premises	64 (32.5%)	133 (67.5%)	76 (46.3%)	88 (53.7%)	7.88	2	0.02
Do not want my place of work to be next door to a psychiatric ward	92 (46.7%)	103 (52.3%)	100 (61.0%)	64 (39.0%)	9.66	4	0.047
Accept location of psychiatric wards within hospital premises	166 (84.3%)	31 (15.7%)	109 (66.5%)	54 (32.9%)	16.5	4	0.00

*P *≤ 0.05 is significant.

df = degrees of freedom.

More male (129 (65.5%)) recipients than females (84 (51.2%)) agreed to other patients not being in danger by locating psychiatric wards within the hospital (χ^2 ^= 8.23, degrees of freedom (df) = 2, *P *< 0.05). The female recipients were also more agreeable to resisting the location of psychiatric wards within the hospital, and to such location posing a great risk to others in the hospital.

The proportion of the female recipients who favoured locating psychiatric wards outside the hospital premises was more than that of the male recipients of the same opinion. Likewise, the female recipients were less keen on having their place of work next door to the psychiatric wards.

A significant number of the male recipients agreed to accepting the location of psychiatric wards within the hospital than the female recipients, (Table 2).

**Table 2:** Crosstabulation report between profession of respondents and responses on 'not wanting place of work next door to psychiatric wards' by the respondents

Profession	Agree (%)	Disagree (%)	χ^2^	df	*P *value
Medical doctor	37 (48.1%)	40 (51.9%)	32.47	16	0.009
Nurse	74 (56.1%)	58 (43.9%)			
Pharmacist	3 (37.5%)	5 (62.5%)			
Administrator	30 (61.2%)	19 (38.8%)			
Laboratory scientist	16 (47.1%)	16 (47.1%)			
Social worker	2 (40.0%)	3 (60.0%)			
Physiotherapist	2 (33.3%)	4 (66.7%)			
Medical records officer	6 (30.0%)	14 (70.0%)			
Support staff	22 (71.0%)	9 (29.0%)			

*P *≤ 0.05 is significant.

df = degrees of freedom.

More than three in every five of the hospital's nurses, administrators and support staff significantly did not favour having their place of work next door to the psychiatric wards. A little more than half of the doctors and more than three in every five of the hospital's pharmacists, social workers, physiotherapists, and medical records officers would not mind having their place of work next door to the psychiatric wards.

More than half of nurses, administrators, support staff, and laboratory scientists favoured workers resisting the location of psychiatric wards within the hospital premises. An equal proportion of physiotherapists were either in favour or against the above opinion. The majority of doctors, pharmacists, social workers and medical records officers that answered the questionnaire were not in favour of the former opinion.

There was a significant relationship between the profession of the respondents and their attitude towards resisting location of psychiatric wards within the hospital premises, (Table 3). Age, marital status, religion, and tribe of the respondents were not significantly related to their attitude to the care of psychiatric patients within the hospital (*P *> 0.05). A focus group discussion with some of the hospital staff, revealed a repeating remark on the need to have the psychiatry departmental building surrounded by high walls, rather than leaving it 'open' like others.

**Table 3:** Crosstabulation report between profession of respondents and responses on 'good reason to resist location of psychiatric wards within the hospital premises' by the respondents

Profession	Agree (%)	Disagree (%)	χ^2^	df	*P *value
Medical doctor	15 (19.5%)	62 (80.5%)	53.30	16	0.00
Nurse	70 (53.0%)	61 (46.2%)			
Pharmacist	1 (12.5%)	7 (87.5%)			
Administrator	28 (57.1%)	21 (42.1%)			
Laboratory scientist	19 (55.9%)	13 (38.2%)			
Social worker	1 (20.0%	4 (80.0%)			
Physiotherapist	3 (50.0%)	3 (50.0%)			
Medical records officer	5 (25.0%)	15 (75.0%)			
Support staff	19 (61.3%)	12 (38.7%)			

*P *≤ 0.05 is significant.

df = degrees of freedom.

## Discussion

Findings from the survey showed that many hospital workers, especially females, expressed anticipatory fears towards letting psychiatric patients obtain admission for treatment within a general hospital setting. This view was very likely caused by many workers not wishing their place of work to be close to the psychiatric wards. In addition, many nurses, administrators, hospital support staff and laboratory scientists would support resisting provision of such an inpatient care facility inside the hospital.

Negative attributions towards psychiatric patients by Nigerian health workers was claimed to be due to deeply rooted negative cultural beliefs and traditional acts that result in a societal dislike for such patients [3]. A community study on attitudes to mental illness in Nigeria showed that more than half of the respondents' thought psychiatric patients could not receive treatment from normal health facilities [4]. This belief is due to the communities' attribution of dangerousness to people with mental illness, because of occasional violent expressions by them. A worrying implication of this negative understanding of people with psychiatric illness is that communities could meet propositions for the rendering of community based mental healthcare with opposition [4].

A study in Kenya claimed that general health workers, even if they were capable of handling psychiatric problems, preferred such patients to be managed by specialist mental health institutions to having them managed in general wards [8].

Some studies have claimed that stigmatising opinions towards people with mental illness are common among all classes of people in Europe and America [9-11]. These expressed negative opinions towards consumers of mental health services occurred despite the majority of the respondents' understanding of biological and environmental factors in the causation of mental illnesses. However, there were differences in the nature and extent of stigma attached to the various psychiatric illnesses by them [9,10].

Stigma, which signifies a mark to show that someone is of a lesser value than others, abounds among health workers in most cultures [12,13]. Society regards someone labelled mentally ill with a stereotyped negative mindset, and this leads to behaviours towards the sick person that worsens his or her burden of illness. Prejudice towards people with mental illness arises based on the societal ignorance of such persons being dangerous and unpredictable, less competent and unable to live productive lives [12].

Because of the discriminatory behaviour portrayed by societies towards people with mental illness, they tend to become prejudiced towards themselves [14]. In the context of most African societies, this internalised self-dislike brings about denial of illness symptoms and refusal to seek appropriate treatment on time. Both the mentally ill and their relations then make a defensive attribution of the sickness to the influence of supernatural forces, or the handiwork of evildoers. In addition, the above defensive attribution brings about the mentally ill and their relations' preference for solutions in the hands of religious and traditional healers rather than going to hospital.

By the time, both the mentally ill person and his/her relations, who by courtesy and association share this stigma, seek orthodox care, especially in the general hospital setting, they face rejecting attitudes by the health workers. This negative attitude by health workers leads to their inability to detect comorbid physical illnesses in psychiatric patients. In cases where these are detected, the patients are reluctantly and inappropriately cared for.

Stigma of mental illness remains high among health professionals in general hospitals despite having 5% of patients presenting at emergency departments with psychiatric problems, and a high rate of medical and surgical patients with psychological morbidity [15]. The absence of synthesis in the minds of general hospital health professionals about the mind not been separate from the body, and that either could influence the other, was claimed to be a significant contributory factor to their stigmatising attitude to mental illness [16].

It is commendable that despite the health workers fear intentions towards admitting psychiatric patients within the hospital, there were remarkably favourable expressions of attitudes for most of the variables assessing fear and exclusion towards the same persons. The expectation is that people working in health facilities should be more humane towards the sick than others in society would be, and most of the variables not overtly making the respondents feel they would be in too close contact with psychiatric patients may possibly have influenced the choice of response. In addition, that three in every five workers favoured not placing psychiatric wards outside the hospital is an indication of their expressed wish to have psychiatric patients treated within the premises, but in a manner that would mean others around would feel protected from their perceived risk of harm.

The finding that three in every five of the hospital nurses, administrators, and support staff were more rejecting towards having their place of work close to the psychiatric wards might be due to their holding more inappropriate information and deep rooted fears about the dangerousness and unpredictability of such persons than others.

Psychiatric illness is universally equated with violence [1,3,17], and as a result health workers, especially in general hospitals, tend to favour segregation of psychiatric patients, as was found in this study. This negative attribution especially by nurses towards psychiatric patients is likely due to their experiences of aggression, mainly from patients suffering paranoid schizophrenia, to their person [18,19]. In addition, unconsciously stigmatising psychiatric patients by health workers is a psychological compensatory justification for avoidance of someone who is somehow different [3].

Generally, not all types of mental illness are associated with violence, and further most psychiatric patients with illnesses that have a risk of violence are not violent [20]. The risk of violence in a psychiatric patient increases with the type of illness, use of alcohol and drugs, treatment non-compliance, and to some extent lack of insight into illness by the patient. Other factors include strange experiences (such as command hallucinations and paranoid beliefs of persecution), poverty, homelessness and a history of violence and criminality.

People with psychiatric illness are more likely to be the victims, rather than the perpetrators, of violence [17]. Most crimes committed are in no way related to mental illness, but there abound instances of sexual violations on female psychotic vagrants, abuse of vagrants for money making and other rituals, repeated verbal and physical assaults, and unfortunately the death of some vagrants at the hands of members of society. Most of these inhuman experiences are due to societal prejudice and inappropriate aggressive responses towards psychiatric patients.

Violent behaviours by psychiatric patients are most often towards family relations, caregivers and friends rather than strangers. Unfortunately there is no data known for Nigeria on 'population attributable risk percentage' (PAR %) that would have highlighted the percentage of violence attributable to psychiatric illness.

This study showed that more than 60% of the respondents preferred having wards for the treatment of psychiatric patients to those for immune-depressed patients. The public's erroneous beliefs about HIV transmission [21] is a possible explanation for the above finding.

Findings from this study would need repetition in other similar hospitals before generalisation. Some of the hospital health workers could have given responses that are reflective of their status and not actually their true disposition to mental illness.

There is the need for proper harnessing of radio, television and newspapers, which are important sources of information to the public, for dissemination of messages that would help to reduce stigma of mental illness. Psychiatric staff in general hospitals should work towards challenging the portrayal of stigma against their patients by other healthcare providers within and outside the hospitals in which they work. The psychiatric units should be skilled in the proper management of patients that appear violent, to abate situations that might heighten fears in the minds of other members of the hospital community. Mental health services should be properly funded by institutions, government, and policy makers.

In conclusion, although psychiatric patients can be aggressive under some situations, the amount of violence attributable to this scenario is very small. Enlightenment programmes and encouraging friendly interactions with recovering psychiatric patients by healthcare providers are positive approaches towards reducing their prejudices toward psychiatric patients.

## Competing interests

The author declares that they have no competing interests.
